# Sheehan Syndrome: An Unusual Presentation Without Inciting Factors

**DOI:** 10.1089/whr.2019.0028

**Published:** 2020-08-24

**Authors:** Ramya Sethuram, Daniel S. Guilfoil, Renee Amori, Julia Kharlip, Karen M. Berkowitz

**Affiliations:** ^1^ Department of Obstetrics and Gynecology, Drexel University College of Medicine, Philadelphia, Pennsylvania, USA.; ^2^ Division of Reproductive Endocrinology and Infertility, Department of Obstetrics and Gynecology, Wayne State University School of Medicine, Detroit, Michigan, USA.; ^3^ Department of Medicine, Drexel University College of Medicine, Philadelphia, Pennsylvania, USA.; ^4^ Division of Endocrinology, Diabetes and Metabolism, Department of Medicine, Perelman School of Medicine, University of Pennsylvania, Philadelphia, Pennsylvania, USA.; ^5^ Department of Biochemistry and Molecular Biology, Drexel University College of Medicine, Philadelphia, Pennsylvania, USA.

**Keywords:** Sheehan syndrome, postpartum headache, hypopituitarism, agalactia, postpartum amenorrhea, case report

## Abstract

***Background:*** Sheehan syndrome (SS) is a rare complication of severe postpartum hemorrhage or hypotension during the processes of labor and delivery that results in ischemic pituitary infarction and necrosis. In this case report, we describe an unusual presentation of SS without inciting factors.

***Case Presentation:*** A 30-year-old multiparous woman presented 2 hours after a normal spontaneous vaginal delivery with a profound severe headache, and subsequent agalactia, dry skin, and mood changes. She was managed conservatively until 10 months postdelivery when she complained of persistent symptoms including amenorrhea. A brain magnetic resonance (MR) with pituitary imaging revealed findings consistent with SS. The patient's symptoms improved and ultimately resolved after levothyroxine, estrogen replacement therapy, and hydrocortisone were instituted.

***Conclusions:*** SS can present without recognized inciting factors. During the initial phase, women may present with profound headache and/or visual disturbances warranting neurological evaluation. A high index of suspicion and a brain MR with pituitary imaging should prompt early consideration of SS to aid in the diagnosis.

## Background

Sheehan syndrome (SS), first recognized in 1937, is partial or complete postpartum hypopituitarism caused by pituitary infarction and necrosis.^1^ SS usually occurs as a complication of massive postpartum hemorrhage or severe hypotension during or after labor and delivery.^2^ Improved management of severe postpartum hemorrhage has decreased the incidence of SS significantly in the developed world.^3^ According to a large retrospective study, the current incidence in the developed world is 5.1 per 100,000 women.^3^ However, it continues to be reported widely in undeveloped or developing nations where massive postpartum hemorrhage is still a common occurrence.^4^

The pathophysiology of SS has been postulated to be secondary to hypercoagulability associated with pregnancy and the resulting thrombosis of the pituitary arteries, vasospasm of the pituitary arteries secondary to severe hypotension after an epidural and/or delivery, or possible pituitary autoimmunity during pregnancy and postpartum.^5^ Here we report an atypical case of SS in which postpartum hypopituitarism occurred without discernable inciting factors in a patient having neither postpartum hemorrhage nor hypotension during or after labor and delivery.

## Case Presentation

A healthy 30-year-old woman, gravida 3 para 1, presented in active labor at 38 weeks 5 days gestation. Her pregnancy was significant for gestational diabetes mellitus (GDM) controlled by glyburide. She had gestational thrombocytopenia with the lowest recorded platelet count of 111 × 10^9^ per liter with no clinical sequelae. Her body mass index was 22.5 kg/m^2^. Her obstetrical history was notable for three spontaneously conceived pregnancies. A previous pregnancy was significant for GDM, managed with glyburide, followed by an uncomplicated labor and vaginal delivery of a healthy term infant. She also had a prior first trimester spontaneous abortion without complications. She had menarche at age 11 years with normal puberty and sexual development. Of note, at age 3–4 years, she was diagnosed with idiopathic short stature because her growth curve plateaued, but an endocrine evaluation was normal. She was treated with recombinant growth hormone (GH) beginning at 7 years of age, which was discontinued at the onset of puberty at age 12. She reached a final adult height of 152.4 cm (5 feet).

In the current case, the patient had an uneventful labor course and no analgesia or epidural anesthesia. She delivered a healthy term infant by normal vaginal delivery after an active phase of 2 hours, and a rapid second stage of approximately 1 minute duration. She remained normotensive with a blood pressure of 110/59 mmHg–119/62 mmHg and a heart rate of 80–100 beats per minute intrapartum and postpartum. Her total estimated blood loss was 150 cc and immediate postpartum assessment revealed her uterine fundus to be firm after a 7-minute third stage. Two hours postpartum, she experienced an intense bilateral, retro-orbital, and frontal headache, which radiated to her occiput and was described as “a band” around her head. The headache was worse with head movements, especially with bending forward, and she also complained of photophobia.

The patient had no history of headaches including migraines. Physical examination revealed her to be alert, awake, and oriented with normal vital signs and no gross neurological abnormalities. Her pain was temporarily ameliorated by a combination of acetaminophen, diphenhydramine, and metoclopramide. She reported that her pain decreased “from 9 to 3” on a scale of 0 to 10. However, 2 hours later, the pain returned and was severe again. The neurology service was consulted and a thorough neurological examination was normal. A computed tomography (CT) of the head was obtained and revealed no intracranial pathology or hemorrhage and a prominent pituitary gland measuring up to 1.1 cm, which was considered to be physiologically normal during pregnancy. After delivery, she had produced colostrum for the initial 2 days postpartum, but otherwise no milk production from either breast despite extensive pumping. A lactation consultation was sought and the patient was instructed to hydrate better to promote increased breast milk production. She was diagnosed with new onset hormonal migraine and discharged home on postpartum day 2 with prescriptions for diphenhydramine, acetaminophen, and metoclopramide as needed for pain.

She presented to the obstetrician's office on postpartum day 6 complaining of a mild headache and no milk production. She noted this to be distinctly different from her previous delivery, when she had produced abundant breast milk. A serum prolactin was drawn and found to be 12.90 ng/mL, which was deemed to be low for breastfeeding. Metoclopramide 10 mg twice daily was prescribed to increase milk production, but at a 2-week follow-up visit, she was still agalactic and a repeat serum prolactin was 9.7 ng/mL. Over the next several weeks, she developed recurrent headaches, dry skin, constipation, hot flashes, decreased libido, and vaginal dryness. She also reported fatigue, apathy, mood swings, and slow hair growth. She was anorectic and lost 15 pounds over 3 months.

**FIG. 1.**
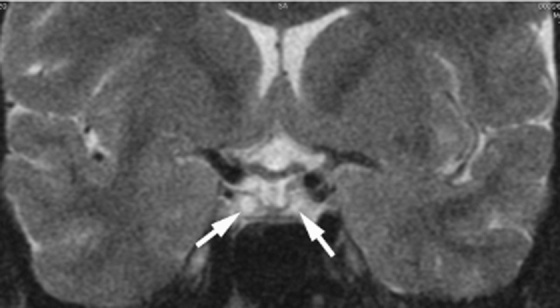
Brain magnetic resonance T2 coronal image illustrating abnormal hyperintense areas of the pituitary gland (arrows).

Menses had still not resumed by 10 months postpartum and she was referred for endocrinologic evaluation. A brain magnetic resonance (MR) performed with gadolinium contrast revealed a normal appearing brain but a 2–3 mm size atrophic pituitary with a diffuse hyperintense T2 signal, consistent with prior injury or infarction (Fig. 1). A serum prolactin was in the low normal range. Serum free thyroxine (T4) was low at 0.49 ng/dL with thyroid stimulating hormone (TSH) only 3.29 mIU/mL. Serum estradiol was low at <5 pg/mL, and luteinizing hormone (LH) and follicle stimulating hormone (FSH) were in the low normal range. An early morning serum cortisol was 8.4 mcg/dL, with adrenocorticotropic hormone (ACTH) 18.2 pg/mL. Serum insulin-like growth factor 1 (IGF-1) was low at 68 ng/mL. A diagnosis of SS was made and levothyroxine 50 mcg daily and an estradiol transdermal patch 0.1 mg/24 hours twice weekly were prescribed initially. Then levothyroxine was increased to an optimal dose of 75 mcg daily and micronized progesterone 200 mg daily for the first 12 days of each month was added after the first month of estradiol treatment.

Her vasomotor symptoms and insomnia resolved and menstrual periods resumed after the first month of therapy. Her anorexia and weight loss, and hair and skin changes improved and ultimately resolved. However, she complained of persistent fatigue and lethargy, and a glucagon stimulation test (GST) revealed no rise in serum GH and a blunted increase in serum cortisol (Table 1). Despite adding GH 0.2 mg daily to her regimen, she continued to feel poorly, so hydrocortisone 15 mg daily was begun with instructions for stress dosing. She had a brief period of polyuria and polydipsia. Daily monitoring of her urine specific gravity revealed an average of 1.018. A serum sodium level was 140 mmol/L, urine osmolality was 576 mOsm/kg, and serum osmolality was 293 mOsm/kg (all normal). These symptoms improved and resolved by 18 months postdelivery. She was advised to start weight-bearing exercise at least three times weekly to preserve her bone mineral density.

**Table 1.** Response to Glucagon Stimulation Test

Serum analyte (reference range)	Baseline value	30 minutes	60 minutes	90 minutes	120 minutes	150 minutes	180 minutes	210 minutes	240 minutes
Glucose (70–99 mg/dL)	84	79	130	64	61	116	85	69	71
Cortisol, mcg/dL^a^	5.9	5.0	5.4	4.1	12.1	9.3	4.6	4.8	9.5
Growth hormone (2.5–3 ng/mL)	<0.1	<0.1	<0.1	<0.1	<0.1	<0.1	<0.1	<0.1	<0.1

Serum values of a 4-hour glucagon stimulation test after a dose of 1 mg glucagon.

^a^ Cortisol of 8.8–22 mcg/dL excludes adrenal insufficiency depending on the study.^18,19^

## Discussion and Conclusions

This case highlights the potential difficulties in diagnosing SS, especially when it presents in the absence of inciting factors such as hemorrhage or hypotension. The lack of diagnostic pointers, when compounded with the broad spectrum of presentation of SS, served as major roadblocks in the path to diagnosis. In this case, our patient complained of acute onset of a headache 2 hours postpartum. The differential diagnosis of postpartum headache includes the most common types such as migraine and tension headache. Other causes of severe headache that are specific to the postpartum phase include pre-eclampsia, postdural headache, meningitis, cerebral venous sinus thrombosis, and acute pituitary apoplexy.^6,7^ Our patient's headache was managed as a migraine in the initial postpartum period and a diagnosis of SS was not considered in the differential until almost a year later when she presented with continued amenorrhea and persistent constitutional symptoms. The delay in her diagnosis was likely due to a lack of initial inciting factors. SS has not been described as a common cause of postpartum headache and hence is generally not included in the initial differential diagnoses.

The literature reports three cases of SS presenting with headache.^8–10^ In one case, the patient presented 5 hours postpartum after an episode of severe hypotension.^8^ The other two cases reported initial presentations with headache 8 hours postdelivery secondary to postpartum hemorrhage in one case and after a delivery complicated by both hypotension and postpartum hemorrhage in the other case.^9,10^ In the first two instances, SS was not considered until patients presented with agalactia and other endocrine symptoms. The third case, complicated by postpartum hemorrhage and hypotension, was diagnosed on postpartum day 6 after readmission because of intense headache, agalactia, dizziness, fatigue, nausea, and vomiting.^9^ In this case, a brain MR with gadolinium contrast revealed findings consistent with pituitary infarction that ultimately led to a diagnosis of SS.

In contrast to these cases, our patient had no intrapartum or postpartum hypotension or hemorrhage, both of which have been identified as clear risk factors in the majority of cases.^5,8–11^ Typically, SS presents after massive postpartum hemorrhage as the inciting event.^5,8–11^ However, SS can rarely present after massive nonobstetric bleeding including brain hemorrhage, intra-abdominal causes, severe trauma, or acute hemorrhagic fever.^5^ SS has also been reported in cases without any antecedent hemorrhage or hypotension, such as anaphylactic shock or liver failure.^5^ Therefore, our case is unique because the patient had no history of hemorrhage, liver failure, or anaphylactic shock as inciting factors.

Interestingly, our patient had a history of idiopathic short stature treated with recombinant GH. Although her endocrine evaluation during childhood was normal and her CT scan immediately postpartum revealed a normal appearing pituitary gland, her history of idiopathic short stature might suggest a predisposition to pituitary dysfunction. However, we did not find this association in the literature.

**FIG. 2.**
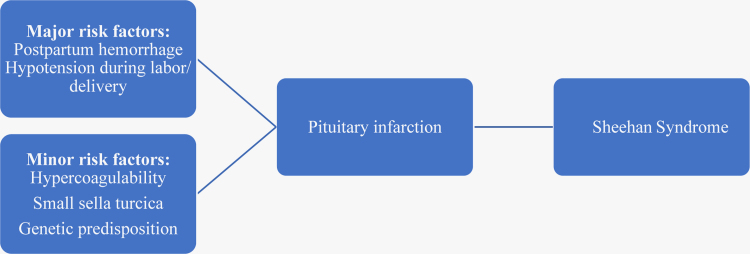
Sheehan syndrome inciting factors.

Although major obstetric antecedents of SS include massive obstetric hemorrhage in one-third of patients and hypovolemic shock necessitating blood transfusion, rare recognized causes of SS include hypercoagulability, small sella turcica, and possible genetic predisposition^5^ (Fig. 2). A congenitally small sella turcica in conjunction with an enlarged pituitary gland of pregnancy can cause compression of the hypophyseal arteries in the sella turcica, resulting in pituitary infarction. Lymphocytic hypophysitis is an autoimmune disorder of the pituitary gland, which can impair pituitary hormonal secretion and is a less frequent cause of postpartum hypopituitarism.^12,13^ Marked lymphocytic infiltration characteristic of this disorder can be suggested by MRI findings, including pituitary enlargement with displacement of the optic chiasma and thickening of the pituitary stalk.

Lymphocytic hypophysitis is also associated with other autoimmune diseases, such as Hashimoto's thyroiditis, autoimmune polyglandular syndrome type 2, Grave's disease, and systemic lupus erythematosis.^12,13^ Our patient did not have any of these associated clinical disorders or radiological findings, and hence, lymphocytic hypophysitis was not considered to be the cause of her hypopituitarism. She did have significant difficulty with lactation compared to her prior pregnancy. She produced only minimal colostrum and subsequently became agalactic; this finding should have prompted further evaluation. She also complained of multiple symptoms including headaches, apathy, vaginal dryness, and dry skin, which were considered to be constitutional and not investigated further. Commonly, SS is diagnosed at a much later time point than the initial presentation of symptoms. In fact, the mean interval between an inciting factor and diagnosis is 13 years (range 16–43 years).^14,15^

About 50% of patients present initially with nonspecific complaints of fatigue, cold intolerance, and sparser hair growth.^14^ These generalized symptoms are known to contribute to the diagnostic delay of SS because they are often mistaken for symptoms of postpartum depression, similar to our patient reported here. Often, SS is diagnosed when postpartum amenorrhea and agalactia are investigated.^15^ Brain MR is the diagnostic test of choice to confirm SS because it provides greater resolution and more detailed findings than other radiological tests such as CT.^16^ Pituitary size is known to increase during pregnancy and the immediate postpartum period.^17^ A partial empty or empty sella turcica are late characteristic features that aid in the diagnosis, when it is not made immediately postpartum.

At the time of SS diagnosis, our patient was found to be hypothyroid and hypoestrogenic. Thyroid hormone and estrogen replacement therapy improved her symptomatology. However, persistent lethargy and fatigue necessitated a GST, which established a diagnosis of GH deficiency. She was started on GH supplementation, but continued to feel unwell. Although her cortisol response of 12.1 mcg/dL to GST was consistent with hypothalamic–pituitary–adrenal dysfunction by some, but not other criteria (8.8–22 mcg/dL),^18,19^ she was offered a trial of hydrocortisone that brought additional symptom relief, suggesting partial adrenal insufficiency. Approximately 50% of patients with SS develop panhypopituitarism, whereas ∼33% of patients develop only adrenal insufficiency, and the remaining 10%–17% of patients present with just hypothyroidism.^14,15^ Rarely, the posterior pituitary is involved and thus, only 5% of patients present with diabetes insipidus.^20^ Examination findings include absence of axillary and pubic hair, pallor, dry skin, and vaginal atrophy in more than half the patients.^14^ The most common pituitary hormones affected are serum TSH, ACTH, FSH, and LH. Serum prolactin is typically spared in patients with partial hypopituitarism.^14^

In the long term, hypopituitarism remains stable or worsens over time.^14^ Similar to our patient who exhibited gradual increasing requirements for hormone replacement, in about half of patients with SS the course is progressive over years, warranting closer follow-up and greater hormone replacement in the long term. The worsening of symptoms and the development of new symptoms over time have been attributed to autoantibodies generated in response to necrotic pituitary tissue.^21^

In summary, this case makes it evident that although the presence of inciting factors is useful as prompts in early evaluation, SS can occur in the absence of recognized and documented inciting factors. In many instances of SS, patients are treated inappropriately with antidepressants, analgesics, and physical therapy for mood changes and fatigue, which delays diagnosis and prolongs their symptomatology. Notably, acute presentation of SS, although rare, can also occur and has been associated with life-threatening complications.^22^ Finally, unremitting headache in the early postpartum period can be an initial presentation of SS and should be considered in the differential diagnosis. When the headache workup is negative, serial brain MR may be indicated. Regardless, agalactia and prolonged postpartum amenorrhea should prompt a diagnosis of SS until proven otherwise.

## Consent for Publication

Written informed consent was obtained from the patient for the publication of her clinical details and clinical images. A copy of the consent form is available for review by the editor-in-chief of this journal.

## Abbreviations Used

ACTH: adrenocorticotropic hormone
CT: computed tomography
FSH: follicle stimulating hormone
GDM: gestational diabetes mellitus
GH: growth hormone
GST: glucagon stimulation test
IGF-1: insulin-like growth factor 1
LH: luteinizing hormone
MR: magnetic resonance
SS: Sheehan syndrome
T4: thyroxine
TSH: thyroid stimulating hormone
